# Diagnosis of rapidly progressed primary cardiac lymphoma in liver transplant recipient: A case report

**DOI:** 10.3389/fonc.2022.1014371

**Published:** 2022-09-23

**Authors:** Jianghua Li, Qiyun Liu, Quanzhou Peng, Shaohong Dong

**Affiliations:** ^1^Department of Cardiology, Shenzhen Cardiovascular Minimally Invasive Medical Engineering Technology Research and Development Center, Shenzhen People’s Hospital, Shenzhen, China; ^2^Department of Pathology, Shenzhen People’s Hospital, Shenzhen, China

**Keywords:** immunocompromised patient, primary cardiac lymphoma (PCL), case report, MRI, echocardiography, lymph node biopsy

## Abstract

Primary cardiac lymphomas (PCLs) are extremely rare and affect the heart. Patients with PCLs usually have delayed diagnosis and treatment. As a consequence, their prognosis is quite unfavorable, and their median survival is approximately 7 months. Herein, we report a 64-year-old man who underwent liver transplantation, presented with chest pain and exertional dyspnea, developed a huge cardiac mass within 2 months and passed away on day 3 of hospitalization. Histological examination revealed diffuse large B-cell lymphoma (DLBCL), which is a rare cardiac tumor with a poor prognosis. In this case, DLBCL was only detected postmortem. The extension of the mass and its relationship with the heart were explored with non-invasive cardiac imaging. Despite the rarity of DLBCL, it should be considered in the differential diagnosis of cardiac tumors.

## Introduction

Primary cardiac lymphomas (PCLs) are an extremely rare type of non-Hodgkin’s lymphoma (NHL) and account for less than 0.01% of all heart tumors (1). PCLs originate in the heart or pericardium and are often diagnosed postmortem (2).

PCLs are frequently observed in immunosuppressed patients (3). The right heart is more susceptible than the left heart (4), and the presentation of patients can be diverse, including congestive heart failure, cardiac tamponade, pericardial effusions, and even death (4). Diffuse large B-cell lymphoma (DLBCL) is the most common pathological of PCLs. Here, we present a case of PCL diagnosed in a subject who passed away on day 3 of hospitalization. This case will enhance the concept that differential diagnosis of cardiac tumors should be considered in clinical practice.

## Case description

As shown in **Table 1**, a 64-year-old man presented with progressive atypical chest pain and exertional shortness of breath for 2 months. He had a history of liver cancer in 2010 and had undergone liver transplantation. Since then, he had regularly consumed immunosuppressive drugs. In addition, he was diagnosed with hypertension 2 years ago and paroxysmal atrial fibrillation 0.5 years before. He underwent transthoracic echocardiography (TTE), coronary artery computed tomography, left ventriculography, and coronary angiography (data not shown) in another hospital 2 months before the current hospitalization. No remarkable abnormality was found.

**Table 1:** Timeline of the case: The crucial events in this case.

Timeline of the case	
12 years before	Diagnosed with liver cancer, received liver transplantation, and consumed immunosuppressive drugs.
2 months before	Presented atypical chest pain and exertional shortness of breath, and no certain cause was found.
Day 1	Diagnosed with acute heart failure for remarkably increased NT-proBNP concentration, and a negative clinical response was obtained with the intravenously administered diuretic.
Day 2	TTE and TEE were performed; a lobulated, poorly mobile mass was detected; and MRI and lymph node biopsy were performed to evaluate the cardiac mass.
Day 3	The patient underwent hemodynamic collapse and died.

NT-proBNP, N-terminal pro-B-type natriuretic peptide; TTE, transthoracic echocardiography; TEE, transesophageal echocardiography.

No other substantial medical history was reported, and his medication list was as follows: 150 mg/day of irbesartan, 15 mg/day of rivaroxaban, 200 mg/day of amiodarone, and 1 mg/day of tacrolimus. The patient was referred to our hospital for further comprehensive examination due to progressive chest tightness. He denied pleuritic chest pain and had no reports of cough, sputum production, fever, or night sweats.

He had a regular rhythm at a rate of 67 bpm, normal blood pressure (114/80 mmHg), a respiratory rate within normal limits (16 breaths/min), oxygen saturation of 97% in room air, and tympanic temperature of 36.5°C. No remarkable murmur was found in cardiac auscultation. Respiratory and abdominal examinations had no positive findings.

His N-terminal pro-B-type natriuretic peptide (NT-proBNP) concentration was 9,100 pg/ml with a normal renal function. His arterial blood gas test indicated 76 and 30.6 mmHg of PaO_2_ and PaCO_2_, respectively. C-reactive protein increased to 81.8 mg/L (normal value of <5), and his erythrocyte sedimentation rate was 36 mm/h (normal values of 0–15). Hepatitis B surface antigen (HbsAg) and hepatitis B core antibody (HbcAb) were positive. HBV DNA was 4.37 × 10^4^ (<1 × 10^2^), and CA125 was 51.13 U/ml (normal value of <35). His anti-HIV test result was negative. He was diagnosed with acute heart failure because of the high NT-proBNP level, and a negative clinical response was obtained with the intravenously administered diuretic.

**Figure 1:**
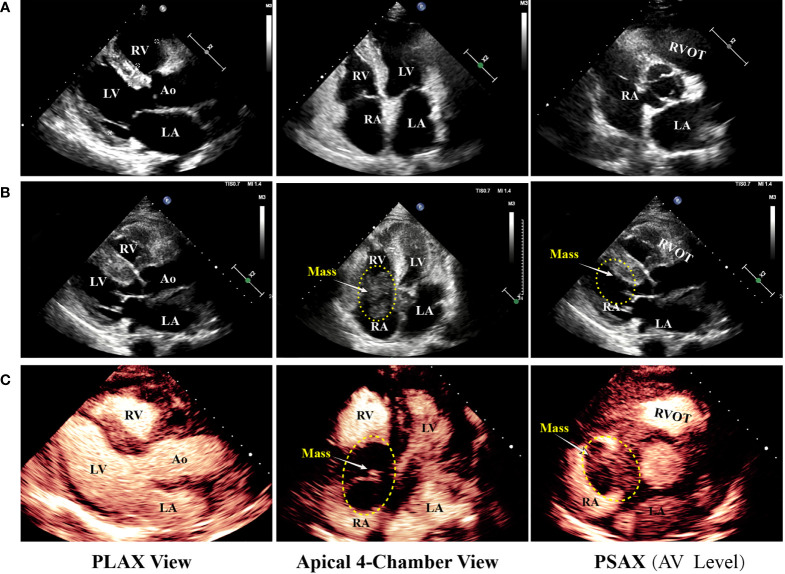
Echocardiography progression of the patient. **(A)** The patient underwent echocardiography 2 months prior to the present hospitalization, and no remarkable abnormality was revealed. **(B, C)** Regular TTE and myocardial contrast echocardiography showing tumor extending from the right atrium to the right ventricle through the tricuspid valve. LA, left atrium; LV, left ventricle; RA, right atrium; RV, right ventricle; Ao, aorta; RVOT, right ventricular outflow tract; PLAX, parasternal long axis; PSAX, parasternal short axis; AV, aortic valve.

**Figure 2:**
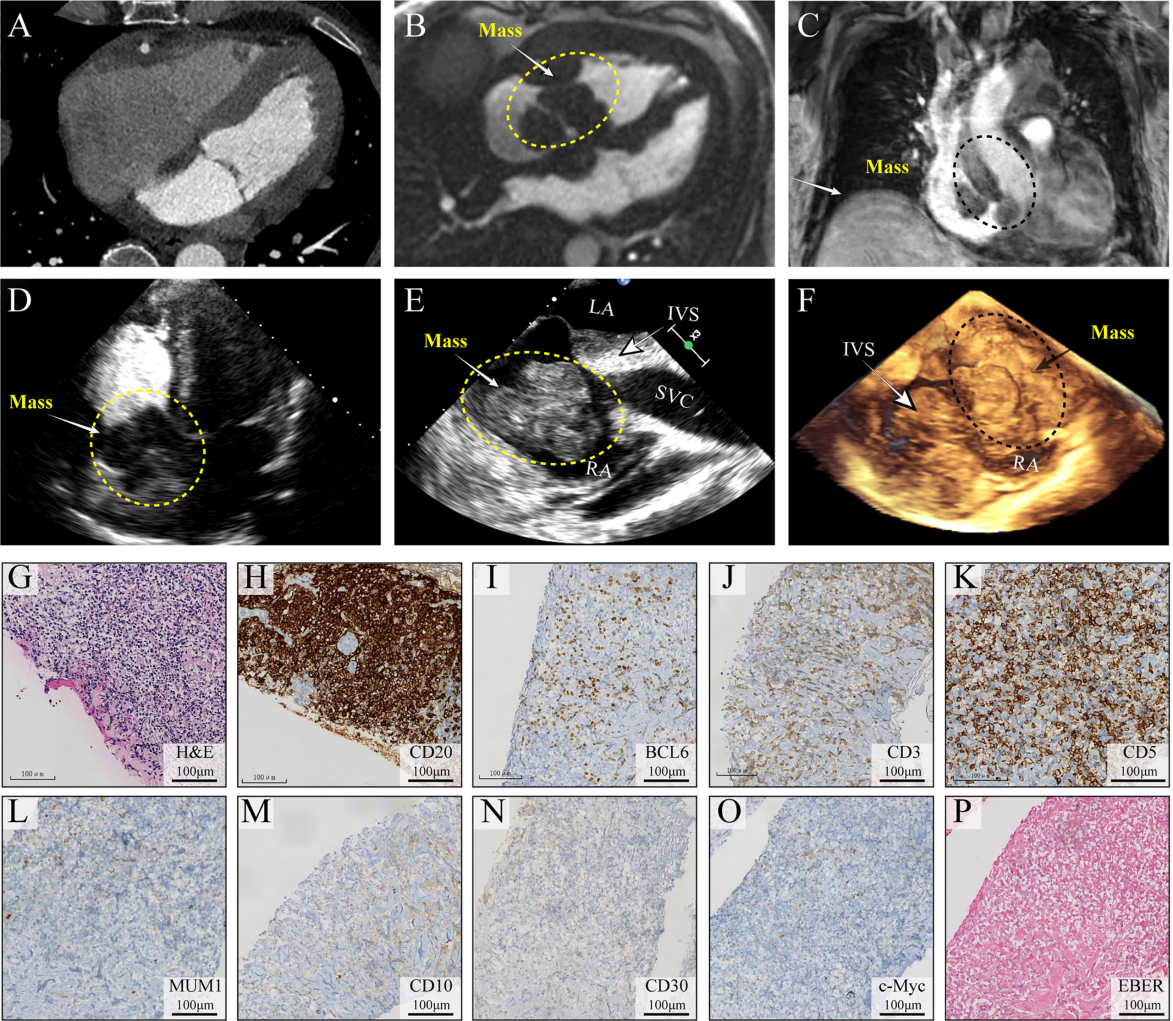
Multimodality medical imaging diagnosis and histopathological examination of the mass. **(A)** Computed tomography obtained 2 months before showed no mass in the right heart. **(B, C)** Cine MRI demonstrated a large infiltrating mass extending in the right atrium and ventricle, occupying most of the right atrium. **(D–F)** Left ventricular opacification, TEE, and 3D ultrasonic imaging of the morphological feature of the mass. **(G–O)** Histological examination of the lymph node, H&E, CD20, BCL6, CD3, CD5, MUM1, CD10, CD30, and c-Myc, **(P)** EBV infection was identified using *in situ* hybridization (ISH) for the detection of EBV-encoded small RNA (EBER) EBER staining. IVS, interventricular septum; TEE, transesophageal echocardiography; EBV, Epstein–Barr virus.

On day 2 of hospitalization, TTE and transesophageal echocardiography (TEE) showed a mass measuring 62 mm × 39 mm originating from the right atrium and right ventricle (**Figure 1**). It was a lobulated, poorly mobile mass that partially occupied the right atrium and partly occupied and occluded the tricuspid, hampering the blood flow to the right ventricle, even though a chest CT scan showed no mass in the right heart 2 months before (**Figure 2A**). In the following magnetic resonance imaging (MRI), the visualization of the mass and its extension improved, demonstrating a large poorly mobile heterogeneous mass obliterating the right atrium cavity. It also revealed the mass infiltration of the surrounding myocardium, with mild pericardial and pleural effusion (**Figures 2B,C**). Myocardial contrast echocardiography and left ventricular opacification revealed that a large filling defect invaded the anterior wall of the right atrium and right ventricle (**Figures 2D-F**). The patient was then subjected to a lymph node biopsy to examine the mass.

Unfortunately, on day 3, before his chest contrast-enhanced computed tomography artery scan was scheduled, he passed away because of hemodynamic collapse. The tissue collected from the lymph node showed a diffuse infiltrate consisting predominantly of large atypical lymphoid cells (**Figure 2G**) and blasts of B-cell lineage (CD20, B-cell marker) (**Figure 2H**); other immunohistochemical pictures, such as BCL6, CD3, CD5, MUM1, CD10, CD30, and c-Myc, were all negative (**Figures 2I-O**). The presence of Epstein–Barr virus (EBV) infection was identified using *in situ* hybridization (ISH) for the detection of EBV-encoded small RNA (EBER) in biopsy tissue samples, which was negative as well (**Figure 2P**). A diagnosis of DLBCL was made (**Figures 2G-I**). In this case, standard biosecurity and institutional safety procedures have been followed.

## Discussion

Our patient is an extremely rare case with a cardiac mass who passed away within 3 days and was diagnosed with PCL postmortem. PCLs are rare cardiac neoplasms that are more frequently found in immunocompromised patients with a median age of 63 years at diagnosis, and the right heart is predominantly affected (3). DLBCL often presents dyspnea, followed by constitutional symptoms and chest pain, and is the most common pathological variant of PCL. Approximately 47% of patients diagnosed with PCL have congestive heart failure resistant to standard heart failure treatment (5).

Multiple cardiovascular advanced imaging modalities can help to morphologically examine the cardiac mass. Echocardiography (**Figures 1**, **2E,F**) is usually the first-line diagnostic test, and its sensitivity in detecting masses is more than 90%. Furthermore, MRI is superior to TTE and TEE in the examination of myocardial and pericardial thickening (1). In addition, a CT scan can help characterize the extent of the mass. For patients with cardiac tumors, the prognosis depends on the type of neoplasms. Patients with PCL have a median survival of 7 months (4), and most patients pass away within a couple of months after diagnosis. Given the severe prognosis of PCLs, early diagnosis and treatment are crucial for these patients.

In our case, 2 months prior to the present hospitalization, the patient was admitted to another hospital and subjected to a CT scan, left ventriculography, and coronary angiography. No remarkable occlusion was detected. However, 2 months later, a large neoplasm in his right atrium was found by TTE, TEE, and MRI. The mass caused cardiac failure and serious hemodynamic abnormality. Unfortunately, the patient underwent hemodynamic collapse and expired on day 3 in our center.

The most common causes of death are intractable heart failure, sepsis, lymphoma progression, arrhythmias, and sudden cardiac death (4). Some potential mechanisms contribute to this unfavorable outcome. First, the obstruction of blood flow and cardiac valve dysfunction may lead to circulation collapse (6); furthermore, arrhythmia is often caused by neoplasm infiltration into the conduction system of the heart. Excessive pericardial effusion usually results in circulatory failure. In the presented case, our patient did not exhibit a notable atrioventricular block or remarkable pericardial effusion but experienced tricuspid valve occlusion and sudden death. Under this condition, traditional diuretics should be cautiously used for this group of patients.

Histopathological examination is the gold standard for the pathological variant of PCL confirmation. More invasive diagnostic procedures, such as cardiac catheterization with echocardiography-guided transvenous biopsy, are required. DLBCL is the most common pathological type of PCL. Its incidence is relatively high in immunocompromised individuals, especially for subjects with AIDS or transplant recipients. In our case, the patient underwent liver translation and received an immunosuppressive agent daily. According to studies that reevaluated reported PCL cases over the past several decades, management has been very variable, and no accepted standard of care has been established (3, 7). According to reports, surgical procedures were given in as many as 29% of the PCL cases that had been investigated (8). However, data from the SEER Program imply that surgical resection is not associated with improved survival in PCL. Chemotherapy was the most common treatment option and was linked to improved survival (9). Consistent with a previous experience, our finding showed an unexpected rapid disease progression. Delayed diagnosis is often correlated with poor outcome because of tumor invasion. Although PCL has no standardized therapy, a chemotherapy regimen with rituximab, cyclophosphamide, hydroxydaunorubicin, vincristine, and prednisone is recommended for this group of patients. Radiotherapy and surgery are given as complementary treatments.

## Conclusions

PCL is a rare cardiac tumor, and its aggressive form is DLBCL. Its early diagnosis and treatment are essential owing to its poor prognosis. Immunocompromised patients are susceptible to this kind of cardiac tumor. The extension of a mass and its relationship with the heart should be explored through non-invasive cardiac imaging. Despite the rarity of DLBCL, its differential diagnosis as a cardiac tumor should be considered.

## Data availability statement

The original contributions presented in the study are included in the article/supplementary material. Further inquiries can be directed to the corresponding authors.

## Ethics statement

This study was reviewed and approved by the Institutional ethics committee of Shenzhen People’s Hospital. The patients/participants provided their written informed consent to participate in this study. Written informed consent was obtained from the individual(s) for the publication of any potentially identifiable images or data included in this article.

## Author contributions

JL wrote the manuscript with support from QL and QP. JL and SD contributed to the conception of the study. All authors contributed to manuscript revision, read, and approved the submitted version.

## Funding

This study was supported by the Medical Scientific Research Foundation of Guangdong Province of China (No. A2018530), Sanming Project of Medicine in Shenzhen (No. SZSM201412012), and Shenzhen Key Medical Discipline Construction Fund (No. szxk003). Shenzhen People’s Hospital Research Cultivation Project (SYLCYJ202119) to JL.

## Conflict of interest

The authors declare that the research was conducted in the absence of any commercial or financial relationships that could be construed as a potential conflict of interest.

## Publisher’s note

All claims expressed in this article are solely those of the authors and do not necessarily represent those of their affiliated organizations, or those of the publisher, the editors and the reviewers. Any product that may be evaluated in this article, or claim that may be made by its manufacturer, is not guaranteed or endorsed by the publisher.
